# Deep Learning–Based Intraoperative Stent Graft Segmentation on Completion Digital Subtraction Angiography During Endovascular Aneurysm Repair

**DOI:** 10.1177/15266028221105840

**Published:** 2022-07-09

**Authors:** Kaj O. Kappe, Stefan P. M. Smorenburg, Arjan W. J. Hoksbergen, Jelmer M. Wolterink, Kak Khee Yeung

**Affiliations:** 1 Department of Surgery, Amsterdam University Medical Centers location, Vrije Universiteit, Amsterdam, The Netherlands; 2 Amsterdam Cardiovascular Sciences, Amsterdam, the Netherlands; 3 Department of Applied Mathematics, Technical Medical Centre, University of Twente, Enschede, The Netherlands

**Keywords:** Deep learning, Artificial Intelligence, Digital Subtraction Angiography, Endovascular Aneurysm Repair, EVAR, Segmentation, Automatic, Intraoperative, Abdominal Aortic Aneurysm, AAA

## Abstract

**Purpose:**

Modern endovascular hybrid operating rooms generate large amounts of medical images during a procedure, which are currently mostly assessed by eye. In this paper, we present fully automatic segmentation of the stent graft on the completion digital subtraction angiography during endovascular aneurysm repair, utilizing a deep learning network.

**Technique:**

Completion digital subtraction angiographies (cDSAs) of 47 patients treated for an infrarenal aortic aneurysm using EVAR were collected retrospectively. A two-dimensional convolutional neural network (CNN) with a U-Net architecture was trained for segmentation of the stent graft from the completion angiographies. The cross-validation resulted in an average Dice similarity score of 0.957 ± 0.041 and median of 0.968 (IQR: 0.950 – 0.976). The mean and median of the average surface distance are 1.266 ± 1.506 mm and 0.870 mm (IQR: 0.490 – 1.430), respectively.

**Conclusion:**

We developed a fully automatic stent graft segmentation method based on the completion digital subtraction angiography during EVAR, utilizing a deep learning network. This can provide the platform for the development of intraoperative analytical applications in the endovascular hybrid operating room such as stent graft deployment accuracy, endoleak visualization, and image fusion correction.

## Introduction

Since its introduction, endovascular aneurysm repair (EVAR) has been inextricably dependent on intraoperative fluoroscopic imaging. Technical innovations in the past decade have led to the replacement of the mobile fluoroscopic C-arm with modern high-tech hybrid operating rooms. With the increased imaging capabilities of the contemporary hybrid operating room, large numbers of fluoroscopy images and digital subtraction angiography (DSA) images are generated during and after the EVAR procedure. Intraoperative clinical decision-making has been predominantly based on visual inspection of images by the operating team, with the exception of computed tomography-fluoroscopy image fusion for navigational purposes.^1,2^ However, while hundreds of images are acquired during a typical intervention, the vast majority of these go unused, while they may have significant value to add information to the procedure or improve procedural outcomes.

The interest in artificial intelligence techniques, and particularly deep learning, has exploded in the past years, driven by an increase in computational power, the utilization of large data sets, and image-guided surgery.^3^ We propose that these techniques can be used to fully exploit all acquired intraoperative images at no additional burden to the operating team. A considerable advantage of using deep learning algorithms is the objective analysis of images versus the subjective visual inspection by the operating team. Moreover, because the number of examples that a deep learning algorithm is exposed to during training is only limited by the availability of data, its exposure could be higher than that of a single observer, potentially outperforming humans. In terms of clinical implementation, this has the potential of improving interobserver agreement during clinical decision-making in the operating room.

Completion digital subtraction angiographies (cDSAs) performed at the end of the procedure after stent graft deployment contain information on the stent graft’s position, possible endoleaks, patency of arteries and stent-graft limbs, and blood flow dynamics. Currently, the cDSAs are assessed by the operating team during the procedure by eye. Since the aforementioned information can be subtle, this important information can be missed by the operating team such as a small endoleak. When this information is known during the procedure and the physician is alerted by the algorithm, extra steps can be performed intraoperatively to prevent reintervention. To fully exploit the possible hidden and valuable information in these images, we propose to analyze these images using deep learning. In this article, we present fully automatic segmentation of the stent graft on a cDSA, utilizing a deep learning network. We hypothesize that an automatic stent graft segmentation method can be developed which can be incorporated into intraoperative clinical applications such as stent graft deployment accuracy analysis during the procedure, endoleak visualization, and image fusion overlay correction, all performed by artificial intelligence–based techniques.

**Clinical Relevance and Implementation**

Completion digital subtraction angiographies (cDSAs) performed at the end of endovascular aneurysm repair (EVAR) contain important information of the stent graft position, patency of arteries and stent graft limbs, and possible endoleaks. All these clinical features are currently assessed by the operating team during the procedure by eye. It is possible that some of these clinical important features can be missed, as some endoleaks are subtle. Also, the position of the stent graft relative to the renal arteries is not assessed in millimeters during the procedure. An artificial intelligence–based algorithm can analyze the cDSA images on a pixel-detailed level, which can be a challenge for humans to see. When this information is analyzed during the procedure, the physician can be alerted by the algorithm and extra steps can be performed intraoperatively to prevent reintervention, such as additional ballooning, placement of a proximal cuff, use of endoanchors, and extension of EVAR limbs. We hypothesize that our fully automated stent graft segmentation method can be incorporated into intraoperative analytical applications such as advanced stent graft deployment assessment during the procedure, endoleak visualization, and image fusion overlay correction, all performed fully automated by artificial intelligence–based techniques.

## Technique

### Data Collection

Completion digital subtraction angiographies of 47 patients treated for an infrarenal aortic aneurysm using EVAR were collected retrospectively. Patients in the study cohort received a bifurcated stent graft, bifurcated stent graft with an iliac branch device, or a single-limb aortoiliac stent graft. The collection of cDSAs from the patients’ electronic medical records was performed with the approval of the local medical ethical review board. All cDSAs were acquired using a C-arm x-ray system in a Philips Azurion FlexMove 7 C20 Hybrid Operating Room (Philips Healthcare, Best, the Netherlands) in the Amsterdam University Medical Centers, location VUmc, in the period from May 2017 to April 2020. Images were acquired with isotropic and anisotropic dimensions ranging from 790 × 1024 to 1904 × 1904 pixels with isotropic pixel sizes ranging from 0.154 × 0.154 mm^2^ to 0.370 × 0.370 mm^2^. The cDSAs were acquired with a multiphase acquisition protocol; phase 1 for 6 seconds at 3 frames per second (fps), phase 2 for 5 seconds at 1 fps, and phase 3 at 0.5 fps indefinitely. Typically, a cDSA contained between 18 and 43 images per series. A purposely induced apnoea minimalized visceral and stent graft motion during the recording of all series.

### Image Modification

We assumed that the stent graft did not move during a cDSA series and therefore posed our segmentation problem as a 2-dimensional (2D) segmentation problem, in which one stent graft segmentation is obtained per cDSA series. To represent a cDSA series as a 2D image, a maximum intensity projection (MaxIP) was obtained along its temporal axis. This resulted in one projection image per cDSA, in which the visibility of the injected contrast agent was minimized, thereby maximizing the visibility of the stent graft. To train and evaluate the deep learning network, pixel-wise manual segmentation masks of the stent graft were created by an expert for all cDSA series to serve as the ground truth. Subsequently, all 2D projection images and corresponding ground truth masks were resampled to 0.4 × 0.4 mm^2^ isotropic pixel spacing using bilinear interpolation and nearest neighbor interpolation, respectively.

### Neural Network Architecture and Training

**Figure 1.**
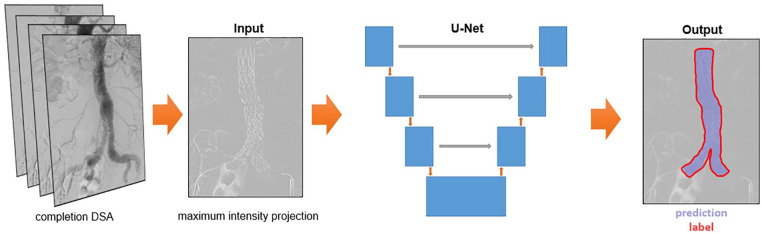
Overview of deep learning-based segmentation method with the maximum intensity projection and ground truth label as input and the corresponding output. The output image has a Dice score of 0.979. DSA, digital subtraction angiography.

A 2D convolutional neural network with a U-Net architecture was trained for segmentation of the stent graft on the 2D projection images, as illustrated in Figure 1.^4^ Training of the neural network was performed using randomly-extracted image patches with size 512 × 512 pixels from the projection images. Training patches were augmented to create additional synthetic data to increase the generalizability of the network. Data augmentation consisted of horizontal flipping with a probability of 0.5 and rotation around the image center with a maximum deviation of ±14°. The network was trained by minimizing a Dice loss with an Adam optimizer algorithm using a learning rate of 0.001, dropout of 0.2, and a batch size of 8. The training was performed for 2000 epochs with a reduction of the learning rate by a factor 10 after 1000 and 1500 epochs.

The network was implemented using the PyTorch-based framework Medical Open Network for AI (MONAI)^5^ and run on a workstation with a NVIDIA GeForce RTX 3090 Graphics Card with 24 GB of memory.

### Evaluation of the Network’s Performance

The performance of the network was evaluated based on a Dice similarity score, which measures the overlap between the predicted segmentation by the model and the ground truth mask. Moreover, we computed the average surface distance between the ground truth and predicted stent graft segmentations. This is the average distance (in millimeters) from all points on the boundary of the predicted segmentation to the closest point on the boundary of the ground truth mask.

**Figure 2.**
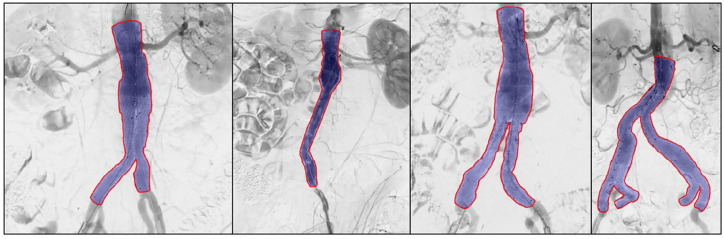
Successful automatic stent graft segmentation results (purple) and corresponding ground truth labels (red). Note that the first three stent grafts have suprarenal fixation and the prediction is based on the stent graft struts and not the fabric. The Dice similarity scores for the shown predictions are 0.985, 0.976, 0.983, and 0.976.

**Figure 3.**
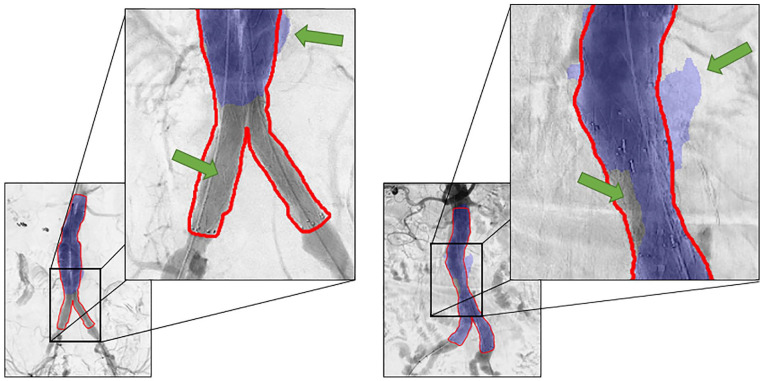
Two patients with suboptimal segmentation of the stent graft. The upper green arrows indicate an area predicted outside the ground truth label (over-segmentation). The lower green arrows indicate under-segmentation. The left prediction has a Dice score of 0.719 and the right prediction has a Dice score of 0.930.

**Figure 4.**
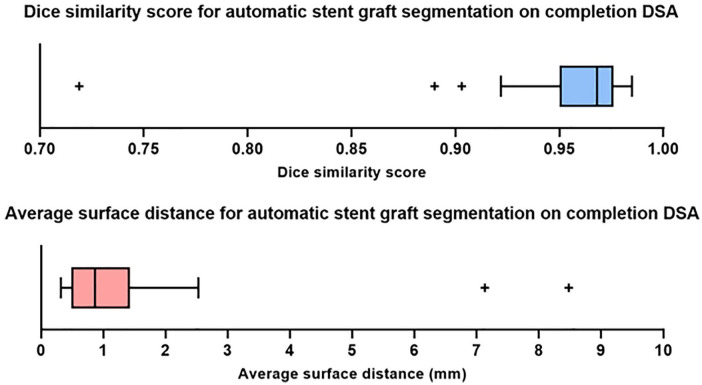
Box plots demonstrating the quantitative results (Dice similarity score and average surface distance) of the cross-validation on the full data set. DSA, digital subtraction angiography.

We performed a 9-fold cross-validation on the full data set. Automatic stent graft segmentations with a high Dice similarity score on unseen test images are shown in Figure 2. The results show that the network is capable of segmenting bifurcated stent grafts, aorto-uni-iliac stent grafts, and bifurcated stent grafts with iliac branch devices, irrespective of the device manufacturer. Moreover, the network is able to correctly annotate stent grafts with infrarenal and suprarenal fixation. Figure 3 shows the result of the largest outlier in Figure 4, together with another suboptimal segmentation as a result of over-segmentation and under-segmentation.

Quantitative results are shown in Figure 4. A Shapiro-Wilk test and Q-Q plots show that both the Dice similarity scores and average surface distances are non-normally distributed. The cross-validation resulted in an average Dice similarity score of 0.957 ± 0.041 and median of 0.968 (interquartile range [IQR]: 0.950-0.976). The mean and median of the average surface distance are 1.266 ± 1.506 mm and 0.870 mm (IQR: 0.490-1.430), respectively. The time to perform the segmentation of a stent graft per patient was on average 1.5 seconds.

## Discussion

The results of our study demonstrate that a deep learning–based method can fully automatically segment the stent graft on cDSA images in 1.5 seconds. The proposed method can provide the basis for further development of clinical analytic applications in the modern endovascular hybrid operating room. This includes objective assessment of stent graft deployment, presence of endoleaks, patency of arteries and stent graft limbs, and blood flow dynamics.

Inspection of the results shows that our proposed method is capable of segmenting different configurations of stent grafts, i.e., bifurcated stent grafts with and without iliac branch devices and aorto-uni-iliac stent grafts. The different strut structures and suprarenal and infrarenal fixation did not seem to influence the performance of the deep learning network.

Several previous studies have reported automatic feature extraction of angiographic images during EVAR. These studies mainly focus on characterization and quantification of arterial deformations after insertion of stiff guide wires, intraoperative correction of image fusion, and automatic segmentation of guidewires.^6–11^ Closest to our work is the study of Breininger et al,^12^ who also proposed a fully automatic deep learning–based method for stent graft segmentation in single 2D fluoroscopic images. Note that in contrast to their method, we perform automatic segmentation on a 2D projection obtained from the entire 2D + time cDSA series. This leads to results that appear to be slightly more accurate than those reported by Breininger et al,^12^ who obtained an average Dice coefficient score of 0.943 ± 0.043, whereas the average of our Dice coefficient was 0.957 ± 0.041.

The study was limited by the number of available cDSAs. Data augmentation was used to partially compensate for the limited number of available imaging. However, expansion of the data set would likely further improve the performance and robustness of the deep learning network. Despite the high Dice similarity scores of our experiment, one major outlier as a result of under-segmentation can be observed in Figure 4 of which the result is visualized in Figure 3. Limited visibility and contrast of the stent graft limbs on the 2D projection may be the underlying cause. Over-segmentation, shown in Figure 3 as well, may be due to substantial background artifacts due to existing contrast agent in the intestines. Additional input channels to the network comprising different 2D projections of the cDSA may help reduce over-segmentation and under-segmentation.

Our proposed method for fully automatic stent graft segmentation lays the foundation for further development of clinical analytical applications for cDSAs to fully exploit possible valuable information. Future work can focus on the development of deep learning networks for segmentation of anatomical structures and analysis of flow parameters. The merging of separate models can lead to the development of fully automatic analytical applications to assist the surgeon in more objective clinical decision-making in the hybrid operation room.

## Conclusion

We developed a fully automatic stent graft segmentation method based on the cDSA during EVAR, using a deep learning network. This can provide the platform for the development of intraoperative analytical applications in the endovascular hybrid operating room such as stent graft deployment accuracy assessment, endoleak visualization, and image fusion correction.
